# Co-Removal Effect and Mechanism of Cr(VI) and Cd(II) by Biochar-Supported Sulfide-Modified Nanoscale Zero-Valent Iron in a Binary System

**DOI:** 10.3390/molecules27154742

**Published:** 2022-07-25

**Authors:** Rui Zhao, Xiufeng Cao, Tao Li, Xiaowei Cui, Zhaojie Cui

**Affiliations:** 1 School of Environmental Science and Engineering, Shandong University, 72 Binhai Road, Jimo District, Qingdao 266237, China; zhao.r@mail.sdu.edu.cn (R.Z.); caoxiufeng@email.sdu.edu.cn (X.C.); litao1699@163.com (T.L.); 2 School of Municipal and Environmental Engineering, Shandong Jianzhu University, 1000 Fengming Road, Lingang Development Zone, Jinan 250101, China; cuixw_1018@163.com

**Keywords:** BC-SnZVI, Cd(II), Cr(VI), co-removal, mechanisms

## Abstract

This study aimed to explore the co-removal effect and mechanism of Cr(VI) and Cd(II) with an optimized synthetic material. The toxicity and accumulation characteristics of Cr(VI) and Cd(II) encountered in wastewater treatment areas present significant challenges. In this work, a rational assembly of sulfide-modified nanoscale zero-valent iron (SnZVI) was introduced into a biochar (BC), and a Cr(VI)–Cd(II) binary system adsorbent with high efficiency was synthesized. When the preparation temperature of the BC was 600 °C, the molar ratio of S/Fe was 0.3, the mass ratio of BC/SnZVI was 1, and the best adsorption capacities of BC-SnZVI for Cr(VI) and Cd(II) in the binary system were 58.87 mg/g and 32.55 mg/g, respectively. In addition, the adsorption mechanism of BC-SnZVI on the Cr(VI)-Cd(II) binary system was revealed in depth by co-removal experiments, indicating that the coexistence of Cd(II) could promote the removal of Cr(VI) by 9.20%, while the coexistence of Cr(VI) could inhibit the removal of Cd(II) by 43.47%. This work provides a new pathway for the adsorption of Cr(VI) and Cd(II) in binary systems, suggesting that BC-SnZVI shows great potential for the co-removal of Cr(VI) and Cd(II) in wastewater.

## 1. Introduction

Heavy metal ion pollution is one of the most serious environmental problems in the field of wastewater treatment, and it has been attracting great attention [1]. Among others, Cr(VI) and Cd(II) are listed as priority pollutants by the US Environmental Protection Agency (EPA) due to their significant toxicity and accumulation properties, which pose a serious threat to ecosystems [2]. In addition, the coexistence of Cr(VI) and Cd(II) in water is a problematic contamination phenomenon, and may cause greater synergistic toxic effects on organisms [3]. Technologies for the removal of heavy metals from water have been explored deeply by researchers, including chemical precipitation, reduction, electrochemistry, and membrane separation [4]. Among them, metal adsorption is more popular and practical, mainly due to its easy operation, low cost, high efficiency, and recyclability [5]. However, Cr(VI) and Cd(II) are different kinds of ions with different charges, so it is not easy for general adsorbents to co-remove these two ions. The co-removal of two different kinds of ions is still a pressing and difficult issue.

Biochar (BC), as a common adsorbent, has caused widespread concern due to its high carbon content, large specific surface area, high cation-exchange capacity, and stable structure [6,7]. Using crop straws such as corn straw as the substrate of biochar is beneficial for waste recycling and green sustainable development [8,9]. However, the limited porosity and functional groups of BC restrict its efficiency for pollutant removal [10,11]. Currently, researchers have found that controlling the preparation process (temperature or ash content) of BC and developing BC-based composites can effectively improve its remediation efficiency. Zhao et al. (2018) found that BC produced from wheat straw at relatively lower pyrolysis showed higher specific surface area, more micropore structures, and higher adsorption capability [12]. Shen et al. (2019) found that magnesium oxide (MgO)-coated corncob BC significantly improved lead removal—from 23.00% to 74.00%—and the surface area of the BC was significantly enhanced from 0.07 to 26.56 m^2^/g after MgO coating [13]. In conclusion, the adsorption capacity of BC could be significantly enhanced by modification.

Sulfide-modified nanoscale zero-valent iron (SnZVI) is a typical nanoscale iron material. It has a core–shell structure and unique physicochemical properties, showing higher electron transfer efficiency compared with nanoscale zero-valent iron (nZVI) [14,15]. However, SnZVI still faces some challenges, such as easy oxidation, agglomeration, and deactivation. BC has a good spatial structure, which could be used as a carrier of SnZVI to prevent the aggregation and oxidation processes. Although several studies on the remediation of heavy metals by BC-SnZVI in water have been conducted [16,17], most studies focused on the removal of heavy metals by BC-SnZVI. There are few studies on the optimization of BC-SnZVI synthesis conditions for better removal efficiency. In addition, some influencing factors in the water environment—such as the coexistence of various ions—also affect the removal of heavy metals by materials. Deng et al. (2020) showed that coexisting anions such as PO_4_^3−^ and NO_3_^−^ inhibited the removal of Cr(VI) [16], but the effect of cations on the removal of heavy metals still needs to be investigated. Importantly, the current research is mainly focused on the removal of single heavy metals [18,19], while only a few studies concern the co-removal of two metals—especially for anionic Cr(VI) and cationic Cd(II). Generally, adsorbents have different removal mechanisms for cations and anions, due to their different charges. For example, the removal mechanism of cationic Cd(II) is generally coordination adsorption [20], while that of anionic Cr(VI) is mainly electrostatic adsorption and reduction [21]. Therefore, specific adsorbents are usually selected for different heavy metals. Furthermore, an antagonistic or synergistic effect between anions and cations could be occur during the co-removal process [22,23]. Based on this, research on the removal of two different types of heavy metal ions by a single adsorbent is a major challenge. Recently, Ahmad et al. (2017) studied the co-removal effect of Cr(VI) and Cd(II) by a chitosan-grafted polyaniline–OMMT nanocomposite [24], but did not address the deep removal mechanism. At present, there is still a lack of research on the co-removal effect and mechanism of anionic Cr(VI) and cationic Cd(II) by BC-SnZVI.

The objectives of this study were to (1) prepare BC-SnZVI with high adsorption efficiency by optimizing the synthesis conditions, (2) explore the removal effect and influencing factors of Cr(VI) and Cd(II), and (3) evaluate co-removal mechanisms of Cr(VI) and Cd(II).

## 2. Materials and Methods

### 2.1. Materials

Corn straws were harvested from local farms. Ferric sulfate (Fe_2_(SO_4_)_3_), sodium borohydride (NaBH_4_), ferrous chloride (FeCl_2_), ferric chloride (FeCl_3_), sodium hydroxide (NaOH), and nitric acid (HNO_3_) were purchased from Shanghai Aladdin Bio-Chem Technology Co., Ltd. (Shanghai, China). All reagents were of analytical grade.

### 2.2. Preparation of BC, BC-SnZVI

The corn straw was pyrolyzed in a muffle furnace (Nabertherm, Frankfurt, Germany) under oxygen-limited conditions, maintained at temperatures of 400, 500, 600, and 700 °C for 4 h, and cooled to room temperature; then, each feedstock was air-dried and milled to pass through a 0.25 mm sieve for further analysis. The BCs produced at different temperatures were named BC400, BC500, BC600, and BC700, respectively.

BC-SnZVI was prepared using the modified method of Zhang [25]. Briefly, 1.89 g of BC produced at 600 °C was mixed with 8.00 g of FeSO_4_·7H_2_O in deionized water, and the mixture was stirred for 30 min. Then, the freshly prepared solution containing excessive NaBH_4_ and 0.75 g of Na_2_S_2_O_4_ was added to the mixture with vigorous stirring under a nitrogen atmosphere for another 1 h. The precipitate was collected and washed with deionized water and ethanol three times. Finally, the resulting precipitate was vacuum-dried at 70 °C. For the BC-SnZVI sample, the molar ratio of S/Fe was 0.3:1, and the mass ratio of SnZVI/BC was 1:1. To optimize the molar ratio of S/Fe and the mass ratio of SnZVI/BC, the molar ratio of S/Fe was varied from 0.2:1 to 0.4:1, and the mass ratio of SnZVI/BC was varied from 1:3 to 3:1.

### 2.3. Batch Adsorption

#### 2.3.1. Screening of Potential Adsorbents

A total of 0.10 g of BC produced at different temperatures or BC-SnZVI was added to 100 mL of K_2_Cr_2_O_7_ or CdCl_2_ solution with a pH of 5.0. The initial concentrations (C_0_) for Cr(VI) and Cd(II) were both 50 mg/L. Subsequently, the flasks were shaken at 25 °C and 160 rpm for 24 h. Then, the samples were filtered through a 0.22 µm membrane filter and characterized. The removal rate of metal ions was calculated by mass balance, as expressed in Equation (1):

Removal rate (%) = 100(C_0_ − C_e_)/C_e_ (1)

where C_0_ and C_e_ are the initial and equilibrium concentrations (mg/L) of metal ions in solution, respectively.

#### 2.3.2. Adsorption in Single and Binary Systems

To investigate the adsorption capacities of screening BC-SnZVI for Cr(VI) and Cd(II) in single and binary systems, 0.10 g of BC-SnZVI was placed in 100 mL of K_2_CrO_4_ or CdCl_2_ solution with a pH of 5.0 and an initial concentration (C_0_) of 10–50 mg/L for both Cr(VI) and Cd(II). The experimental data were analyzed using Langmuir sorption isotherm models according to the method of Sathish et al. (2007) [26].

#### 2.3.3. Effects of the Concentrations of Heavy Metals

To study the effect of metal concentration on competitive adsorption, the concentration of either Cr(VI) or Cd(II) was set to 50 mg/L, while that of the other metal was set to 10–50 mg/L, in a binary system.

#### 2.3.4. Effects of Coexisting Heavy Metals

To investigate the effects of coexisting metal ions (e.g., Pb, Hg, As), BC-SnZVI was added to a 100 mL mixed solution of CdCl_2_, K_2_CrO_4_, and different metal ions, with an initial pH of 5.0. The concentration of every heavy metal was set to 50 mg/L.

### 2.4. Characterization

Scanning electron microscopy (SEM) images were obtained with a ZEISS Merlin scanning electron microscope (Carl Zeiss, Jena, Germany). X-ray diffraction (XRD) spectra of the materials were recorded between 10° and 80° (2θ), at a step size of 0.02°, using a Bruker D8 Advance diffractometer with 40 kV voltage and a 40 mA anode current for the Cu Kα radiation (Bruker AXS, Karlsruhe, Germany). Fourier-transform infrared (FTIR) spectra were collected on a PerkinElmer 1725X FTIR spectrometer (PerkinElmer, Waltham, MA, USA). Surface analyses were performed by XPS (ESCALAB250 Xi, Thermo Fisher Scientific, Waltham, MA, USA) using a monochromated Al Kα source and a spot diameter of 400 μm.

### 2.5. Statistical Analysis

Experiments were conducted in triplicate in parallel. The concentrations of Cr(VI) and Cd(II) were determined by inductively coupled plasma atomic emission spectrometry (ICP-AES) (IRIS Intrepid II XSP, Thermo Elemental, Waltham, MA, USA). The data were analyzed by one-way analysis of variance using the SPSS 11.0 software (IB, Armonk, NY, USA). Significant differences were reported at *p* < 0.05 according to Fisher’s least significant difference (LSD) pairwise multiple comparison test. All of the figures were produced with Origin 2021 and arranged using Adobe Illustrator 2020.

## 3. Results and Discussion

### 3.1. Synthesis and Characterization of Materials

#### 3.1.1. Optimized Synthesis of Materials

**Figure 1:**
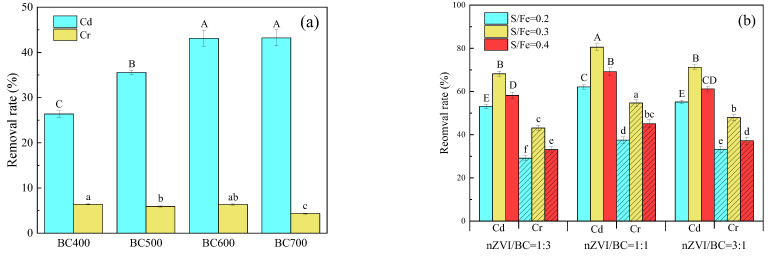
The removal rates of Cd(II) and Cr(VI) by (**a**) BC and (**b**) BC-SnZVI. Different letters on bars indicate significant differences at *p* < 0.05 within the same heavy metal (Cd(II) or Cr(VI)) treatments.

The removal rates of Cr(VI) and Cd(II) by BC produced at different temperatures are shown in Figure 1a. It can be observed that the removal rate of Cd(II) increased gradually with the increase in temperature for producing BC, but it did not increase significantly after 600 °C. The removal rate of Cr(VI) even slightly decreased with the increase in temperature for producing BC. According to Table S1, the H/C rate representing the aromatization structure of BC and the O/C rate representing the polarity of BC decreased with increasing temperature, indicating that higher temperatures could promote the conversion of aliphatic hydrocarbons to aromatic hydrocarbons. With the increase in temperature, the greater the aromatization of BC, the stronger the cation π effect [27]. Furthermore, the higher the temperature, the greater the ash content and specific surface area. These reactions all increased the adsorption of Cd(II) by BC. With the increase in temperature, the acidic functional group (-OH) decreased and the basic functional group (-C-N-) increased (Figure S1, Supplementary Materials), which may be one of the reasons for the weakened adsorption of Cr(VI) by BC [10,28]. Therefore, BC600 was selected as the test material for the next experiment, according to the removal rate and preparation cost.

For BC-SnZVI, the removal rates of Cr(VI) and Cd(II) increased with the increase in the S/Fe molar ratio from 0.2 to 0.3, but decreased slightly with the increase in the S/Fe molar ratio from 0.3 to 0.4 (Figure 1b). This phenomenon could be explained by the fact that the sulfur modification significantly improved electronic selectivity and enhanced the efficiency of electron transfer. However, as the molar ratio of S/Fe further increased to 0.4, the thick iron sulfide layer covering nZVI particle surfaces may have hindered the co-precipitation and adsorption process of heavy metals [15]. The removal rates of Cr(VI) and Cd(II) were the highest when the mass ratio of SnZVI/BC was 1, which could be attributed to the fact that less SnZVI produced an insufficient effect, while more SnZVI would affect the effectiveness of the BC [15,29,30]. When the mass ratio of SnZVI/BC was 1 and the molar ratio of S/Fe was 0.3, the removal rates of Cd(II) and Cr(VI) could reach 80.53% and 54.66%, respectively. The above control conditions were selected for the following study.

#### 3.1.2. Characterization of BC and BC-SnZVI

**Figure 2:**
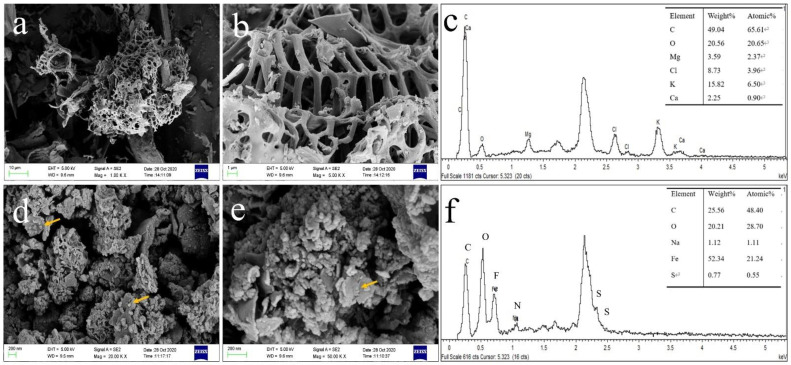
The SEM images of (**a**,**b**) BC and (**d**,**e**) BC-SnZVI; the EDS survey for (**c**) BC and (**f**) BC-SnZVI.

The morphologies of BC produced at 600 °C and of BC-SnZVI are shown in Figure 2a,b,d,e. It can be seen that BC has pore structures to which SnZVI could be attached. It was reported that the SnZVI paraparticles were initially nanoscale, but they were easily aggregated to at least a micrometer scale without surfactants or carriers [16]. According to the SEM images of BC-SnZVI, SnZVI can be dispersed on the surface or pores of BC after loading, and the diameter of SnZVI was less than 100 nm.

The elemental distribution of BC and BC-SnZVI is shown in Figure 2c,f, respectively. Fe and S appeared after loading SnZVI, which also indicated that SnZVI was successfully loaded on the BC. The weight proportions of iron and sulfur in BC-SnZVI were 52.34% and 0.77%, respectively. The atomic proportions of iron and sulfur in BC-SnZVI were 21.24% and 0.55%, respectively.

**Figure 3:**
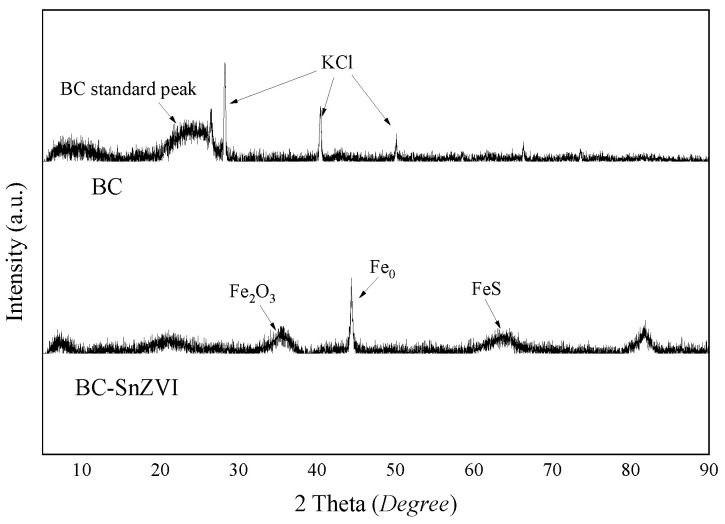
XRD patterns of BC and BC-SnZVI.

The XRD patterns of BC and BC-SnZVI are shown in Figure 3. The spectra of BC showed identical spectra with broad diffraction peaks at 2θ = 24°, which were attributed to the presence of the -OH, O=C-O, and C-O groups [31]. We also found that the sharp 2θ peaks at 28.34°, 40.50°, and 50.16° were assigned to KCl (JCPDS No. 41-1476), which was perhaps because with the increase in temperature, a large amount of salt was dehydrated and precipitated [32]. An Fe^0^ (JCPDS No. 50-1275) peak appeared at 44.66° in BC-SnZVI [18], indicating that ferric or ferrous iron was reduced. The peak of FeS (JCPDS No. 37-0477) was detected at 64.38° [33], suggesting that FeS was synthesized in the loading process. The appearance of Fe^0^ and FeS indicated that SnZVI was successfully synthesized and loaded on BC, which was consistent with the results of SEM. However, the peak of FeS was weak, probably because of the low content and poor crystallinity of FeS [34]. In addition, an Fe_2_O_3_ (JCPDS No. 33-0664) peak also appeared at 35.61° [35], indicating that the zero-valent iron was partially oxidized. The XRD results showed that the synthesized BC-SnZVI was composed of Fe_0_, FeS, and iron oxides.

### 3.2. Isothermal Adsorption in Single and Binary Systems

**Figure 4:**
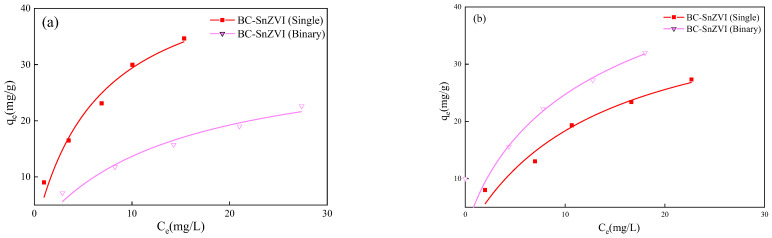
Isothermal adsorption of (**a**) Cd(II) and (**b**) Cr(VI) on BC-SnZVI in single or binary systems.

In order to study the adsorption efficiency of BC-SnZVI, the adsorption isotherms of Cr(VI) and Cd(II) in single or binary systems were evaluated, as shown in Figure 4. The results showed that the maximum adsorption capacities of BC-SnZVI for Cd(II) were 48.65 mg/g in the single system and 32.55 mg/g in the binary system, while for Cr(VI), the maximum adsorption capacities were 42.14 mg/g in the single system and 58.87 mg/g in the binary system. Therefore, Cr(VI) could inhibit the adsorption of Cd(II), while Cd(II) could promote the adsorption of Cr(VI) in the binary system. Chen’s research was partly supportive of this result, finding that Cr(VI) and Cd(II) had a synergistic removal effect in aqueous solution [36]. However, Chen used chitosan and vermiculite as adsorbents, which have different adsorption mechanisms compared to BC-SnZVI. Some studies have shown that when two ions coexist, they compete for the adsorption site of the material, resulting in a decrease in the adsorption efficiency for each ion [37,38]. Zheng et al. (2021) found that coexisting ions have obvious competitive adsorption behavior on chitosan–EDTA-modified magnetic BC when the metal concentration is beyond 20 mg/L [39]. However, some studies also showed that the cations in the solution may increase the positive charge density of the adsorbents, resulting in enhanced electrostatic adsorption capacity for anions which, in turn, increases the removal rate of anions [40,41]. In short, many factors could affect the removal rates of Cr(VI) and Cd(II), which may be related to the structure and functional groups of the material, the concentration of heavy metals, or the type of heavy metal ions.

### 3.3. Effects of Heavy Metal Concentration on Adsorption

**Figure 5:**
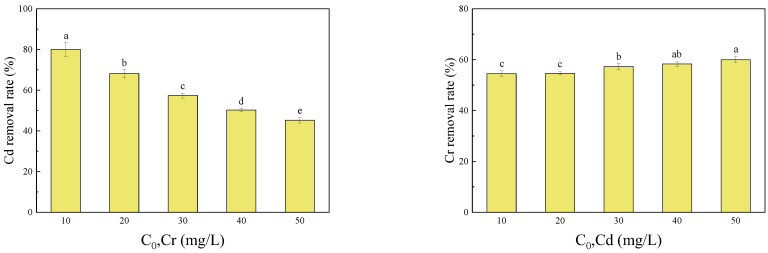
Effects of the initial concentrations of Cr(VI) or Cd(II) on their removal rates in a binary system. Different letters on bars indicate significant differences at *p* < 0.05 between treatments.

As shown in Figure 5, with the increase in the initial Cr(VI) concentration, the removal rate of Cd(II) gradually decreased. This indicated that the Cd(II) adsorption to BC-SnZVI was inhibited by Cr(VI), and the inhibition rate reached 43.47% when Cr was 50 mg/L. However, with the increase in the initial Cd(II) concentration, the removal rate of Cr(VI) gradually increased, showing that the removal of Cr(II) by BC-sZVI was promoted (by 9.20%). The above phenomenon was mainly due to the different adsorption mechanisms of Cr(VI) and Cd(II) by BC-SnZVI. The adsorption mechanisms generally included electrostatic adsorption, redox reactions, coordination adsorption, and precipitation reactions [42,43,44]. The specific reasons should be further discussed with the characterization of materials.

### 3.4. Effects of Other Coexisting Metal Ions

**Figure 6:**
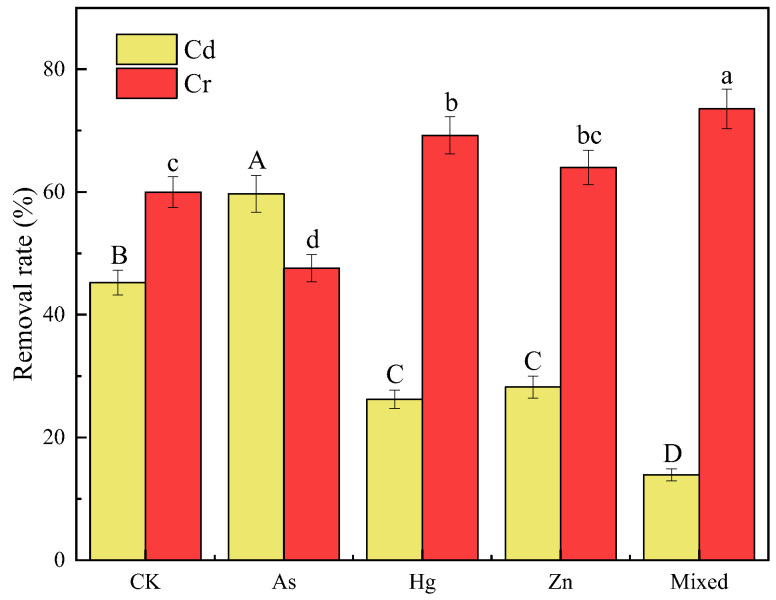
Effects of types of heavy metals on the removal rates of Cr(VI) and Cd(II) in binary systems. Different letters on bars indicate significant differences at *p* < 0.05 within the same heavy metal (Cd(II) or Cr(VI)) treatments.

There are many coexisting heavy metals that could affect the removal of Cr(VI) and Cd(II) in actual environments [45,46]. Therefore, the effects of other heavy metals on Cr(VI)–Cd(II) removal in binary systems were investigated. As shown in Figure 6, the removal rate of Cd(II) could be promoted by the coexistence of As(III) (by 24.22%), but inhibited by the coexistence of Hg(II) and Zn(II) (42.01%, 37.66%). Interestingly, the coexistence of other metals showed the opposite effects to Cr(VI). The removal rate of Cr(VI) was promoted by the coexistence of Hg(II) and Zn(II), and the promotion rates were 13.40% and 6.30%, respectively, while the coexistence of As(III) inhibited Cr(VI) removal by 20.68%. The results showed that there was a competitive adsorption between As(III) and Cr(VI), which may have been due to the site occupation of As(III) to Cr(VI). In addition, the presence of As(III) might reduce the positive charge density of BC-SnZVI, resulting in the decrease in electrostatic adsorption capacity for Cr(VI). Competitive adsorption between Hg(II), Zn(II), and Cd(II) was also found, and the order of competitiveness was Hg(II) > Zn(II). This was mainly attributed to the affinity of these heavy metal ions for BC-SnZVI [46,47].

### 3.5. Comparison with Other Adsorbents

The adsorption capacities of BC-SnZVI and other reported adsorbents were compared, as shown in Table S2. Some adsorbents—such as magnetite NPs, sunflower head carbon, and sunflower stem carbon—could simultaneously remove Cr(VI) and Cd(II) [38,48], but BC-SnZVI had a higher adsorption capacity than these absorbents. Although some adsorbents had better adsorption capacity for Cr(VI) or Cd(II) in single systems, most of them were still lower than that of BC-SnZVI. The results indicate that the optimized BC-SnZVI has a great adsorption effect on Cr(VI) and Cd(II), and show the potential of BC-SnZVI in practical application.

### 3.6. Mechanisms of Cd(II)-Cr(VI) Co-Removal

#### 3.6.1. Effects of Functional Groups

**Figure 7:**
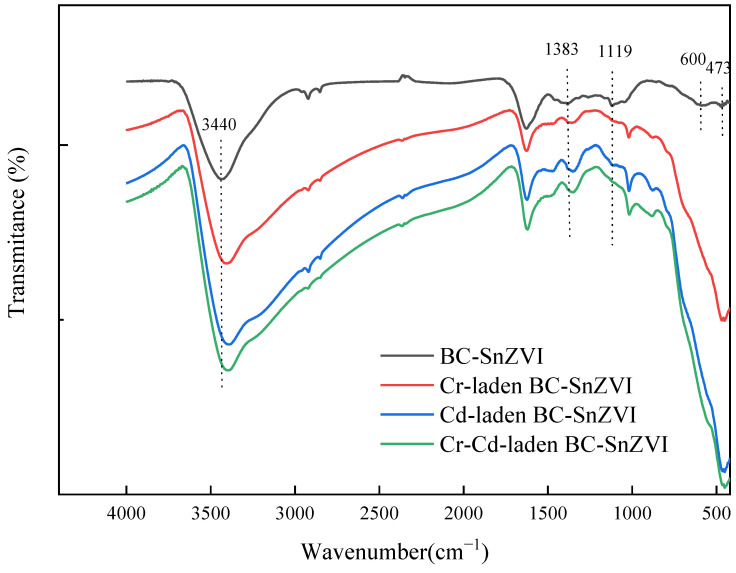
FTIR spectra of BC-SnZVI before and after the adsorption of Cr(VI) and Cd(II).

Studies have shown that BC loaded with SnZVI forms a special C-O-Fe structure, in addition to the common functional groups such as -COOH and -OH. In addition, some iron ions formed iron oxide compounds (yFe_2_O_3_), and some formed Fe-R-COOH and Fe-R-OH functional groups with BC surface groups [49,50]. The assignment of vibrational bands in the FTIR spectra in this study is shown in Table S3. In this study, the peaks of various functional groups of BC-SnZVI changed slightly after Cr(VI) and Cd(II) adsorption (Figure 7). These changes illustrate that various functional groups played important roles in the heavy metal adsorption process. The peak at 3440 cm^−1^ in BC-SnZVI was attributed to the -OH stretching vibration [51], and the peak position was offset after adsorption. The peak at 1383 cm^−1^ was associated with -COOH stretching vibration [52], and the peak vibration was enhanced after adsorption. This indicates the existence of strong interactions between -OH and -COOH groups with Cr(VI) and Cd(II) ions, which is consistent with previous studies [19,25,30]. The change in carboxyl groups was primarily concerned with the reduction of Cr(VI) and the coordination of Cd(II). Hydroxyl groups provided electrons for the reduction of Cr(VI); meanwhile, they were oxidized to carboxyl groups [46,47,53]. Xue et al. (2012) also pointed out that the -COOH functional group played an important role in the adsorption process through coordination adsorption [54]. Meanwhile, the C-O group at 1119 cm^−1^ [30], the Fe-O group at 600 cm^−1^ [19], and the Fe-S group at 473 cm^−1^ [55] for BC-SnZVI were shifted or disappeared after Cr(VI) and Cd(II) adsorption, illustrating that the above groups played a role in the adsorption process.

#### 3.6.2. XPS Analysis

**Figure 8:**
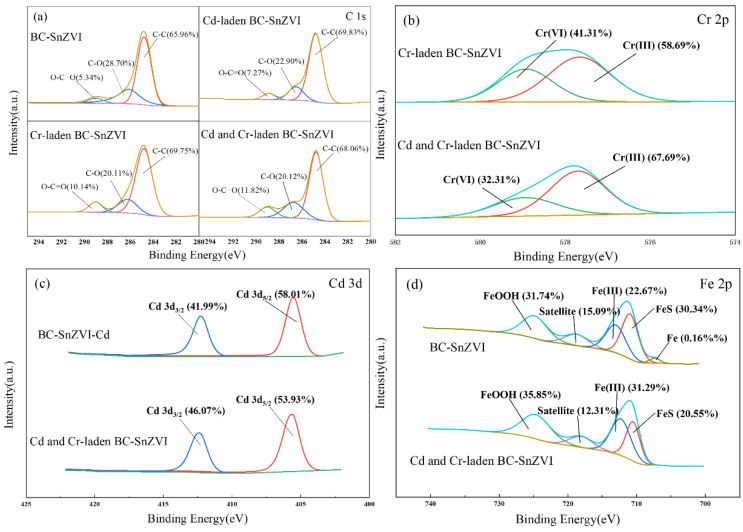
XPS spectra of BC-SnZVI before and after the adsorption of Cr(VI) and Cd(II): (**a**) C 1s, (**b**) Cr 2p, (**c**) Cd 3d, (**d**) Fe 2p.

Figure 8 presents the XPS spectra of BC-SnZVI before and after Cr(VI) and Cd(II) adsorption. From the XPS spectrum of C 1s (Figure 8a), the rate of the O-C=O peak ascended after Cr(VI) and Cd(II) adsorption. Especially in Cr-laden BC-SnZVI and Cr–Cd-laden BC-SnZVI systems, the rate of the O-C=O peak ascended from 5.34% to 10.14% and 11.82%, respectively. However, the C-O peak decreased from 28.70% (BC-SnZVI) to 22.90%, 20.11%, and 20.12% for Cd-laden BC-SnZVI, Cr-laden BC-SnZVI, and Cr–Cd-laden BC-SnZVI, respectively. This was mainly due to the combined results of redox and adsorption. Firstly, the hydroxyl groups were oxidized to carboxyl groups by Cr(VI), resulting in an increase in the rate of the O-C=O peak, which was the same as that reflected in the FTIR. Moreover, it is noteworthy that in the binary system, the rate of the O-C=O peak was larger, indicating that the coexistence of Cd(II) might further promote Cr(VI) reduction. Secondly, oxygen-containing functional groups could coordinate with Cd(II) and Cr(III), resulting in a decrease in the C-O peak rate [56].

From the XPS spectrum of Fe 2p (Figure 8b), BC-SnZVI had an Fe_0_ peak, but the Fe_0_ peak disappeared after ion adsorption. Moreover, the rate of the FeS peak decreased and the rate of the Fe(III) and FeOOH peaks ascended after ion adsorption. This clearly indicated that the redox reaction occurred, and that Fe_0_ and Fe(II) were oxidized after ion adsorption. From the XPS spectrum of Cr 2p (Figure 8c), a part of Cr(VI) was reduced to Cr(III) by BC-SnZVI, and the rate of the Cr(III) peak was higher than that of Cr(VI), indicating that reduction was an important factor for Cr(VI) removal. In addition, the ratio of Cr(III) to total adsorbed Cr was 67.69% in a binary system, which was 9.00% higher than that in a single system. This demonstrates that the coexistence of Cd(II) enhanced Cr(VI) reduction, which is consistent with the results of C 1s. From the XPS spectrum of Cd 3d (Figure 8d), the two peaks of the Cd 3d spectrum did not change noticeably in either single or binary systems, indicating that redox reaction was not the main factor for Cd removal. This was mainly because the standard reduction potential of Cd^2+^ was close to that of Fe^2+^, so the reduction of Cd^2+^ did not occur during the reaction [57].

#### 3.6.3. Simulated Mechanisms

**Figure 9:**
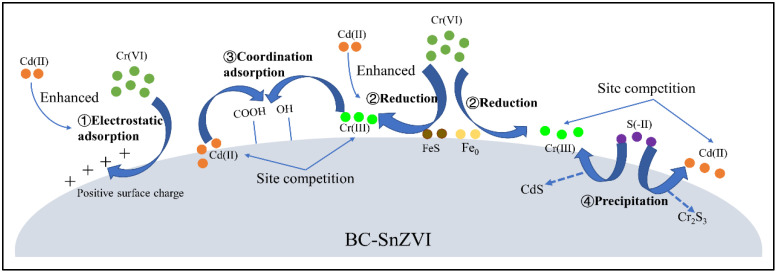
The co-removal mechanism of Cd(II) and Cr(VI) by BC-SnZVI.

The co-removal mechanism of Cr(VI) and Cd(II) is shown in Figure 9. The removal of Cr(VI) was a multiple-reaction process. Firstly, Cr(VI) was adsorbed on the surface of BC-SnZVI by electrostatic adsorption due to the positive surface charge of BC-SnZVI. In addition, the coexistence of Cd(II) improved the positive charge density of BC-SnZVI, and promoted the effect of electrostatic adsorption. Secondly, Cr(VI) was reduced to Cr(III) by Fe_0_, FeS, and the -OH functional group, and the coexistence of Cd(II) further promoted the reduction of Cr(VI). Finally, a part of Cr(III) could form Cr_2_S_3_ precipitation with S^2−^, and a part of Cr(III) could generate coordination adsorption with oxygen-containing functional groups. Therefore, the coexistence of Cd(II) promoted the removal of Cr(VI) in the binary system. Conversely, Cd(II) was mainly removed by coordination adsorption and precipitation reactions, since redox reactions were not involved. On the one hand, Cd(II) could form CdS precipitation with S^2−^. On the other hand, it could generate coordination adsorption with oxygen-containing functional groups. However, the coordination adsorption and precipitation of Cd(II) was weakened due to site competition from Cr(III). Therefore, the coexistence of Cr(VI) could inhibit the removal of Cd(II) in the binary system, and the inhibition was positively related to Cr(VI) content.

## 4. Conclusions and Future Perspectives

This study explored the effectiveness, influencing factors, and mechanisms for the co-removal of Cr(VI) and Cd(II) by BC-SnZVI. The results showed that optimized BC-SnZVI had great effectiveness in the co-removal of Cr(VI) and Cd(II). The adsorption capacity of Cd(II) and Cr(VI) was 32.55 mg/g and 58.87 mg/g, respectively, in the binary system. The coexistence of Cd(II) could promote the removal of Cr(VI) by 9.20%, while the coexistence of Cr(VI) inhibited the removal of Cd(II) by 43.47%. Moreover, the coexistence of As(III) promoted the removal of Cd(II) but inhibited Cr(VI) removal, while the coexistence of Hg(II) and Zn(II) promoted the removal of Cr(VI) but inhibited Cd(II) removal. By exploring the potential mechanisms, the coexistence of Cd(II) promoted the electrostatic adsorption of Cr(VI) by improving the positive charge density of BC-SnZVI. In addition, the reduction of Cr(VI) was also promoted, enhancing the removal of Cr(VI). Meanwhile, the coordination adsorption and precipitation of Cd(II) were weakened due to the site competition of Cr(III). This study could provide theoretical guidance for the co-removal of different types of heavy metal ions in wastewater.

Future research should be carried out from the following perspectives: (1) the co-removal effect of heavy metals can be further enhanced by introducing some functional groups, such as sulfhydryl groups; (2) other substances that have an impact on heavy metal removal should be investigated, including emerging pollutants such as microplastics and antibiotics; and (3) the co-removal effects or mechanisms of other different types of heavy metals should be further studied.

## Data Availability

Not applicable.

## Supplementary Materials

The following supporting information can be downloaded at: https://www.mdpi.com/article/10.3390/molecules27154742/s1, Figure S1: FTIR spectra of BC produced at different temperatures; Table S1: Elemental analysis of BC produced at different temperatures; Table S2: Comparison of the adsorption capacities of Cr(VI) and Cd(II) by different adsorbents; Table S3: Assignment of the vibrational bands in the FTIR spectra. References [58,59,60,61,62] are cited in Supplementary Materials.

## Sample Availability

Samples of the compounds of biochar-supported sulfide-modified nanoscale zero-valent iron are available from the authors.
