# Supplementary material for: Co-Removal Effect and Mechanism of Cr(VI) and Cd(II) by Biochar-Supported Sulfide-Modified Nanoscale Zero-Valent Iron in a Binary System
Source: Molecules. 2022 Jul 25;27(15):4742. doi: 10.3390/molecules27154742 (PMC9331559; doi:10.3390/molecules27154742)
Supplement: Supplementary file 1 [file molecules-27-04742-s001.zip › molecules-1824881-supplementary .pdf]

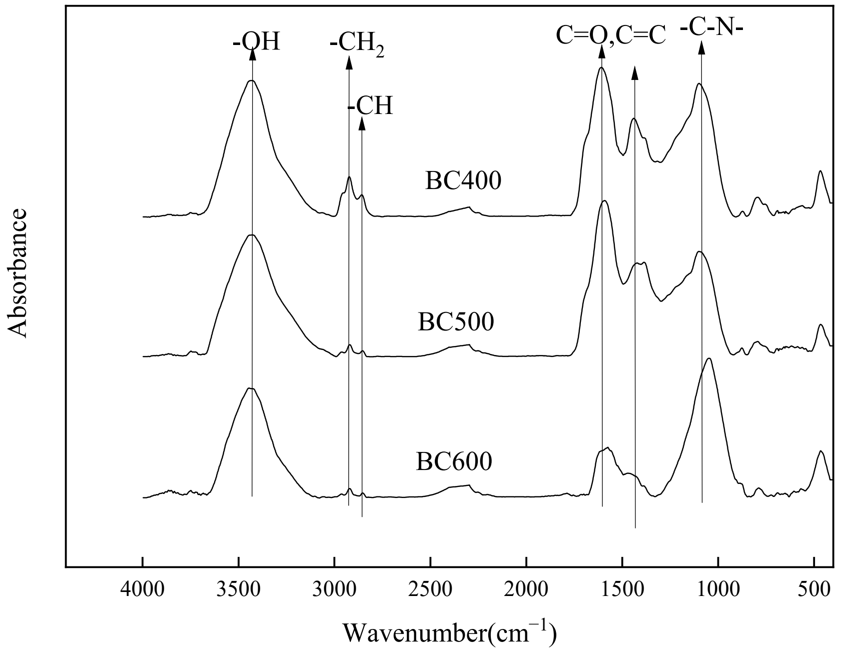

Figure.S1 FTIR spectra of BC produced at different temperatures

Table S1 Elemental analysis of BC produced at different temperatures

Biochar	Elements（%）					Atomic ratio		Ash(%)	Specific surface area (m^2/g)	)pH
	N	C	H	S	O	H/C	O/C			
BC400	1.41	55.20	4.23	0.15	21.30	0.92	0.29	13.12	14.83	7.6
BC500	1.36	67.30	3.21	0.17	16.13	0.57	0.18	16.02	50.41	8.35
BC600	1.17	73.20	1.98	0.18	14.10	0.32	0.14	20.14	80.12	9.78
BC700	1.21	77.60	1.28	0.15	12.02	0.20	0.12	24.13	85.03	10.32

Table S2 Comparison of the adsorption capacities of Cr(VI) and Cd(II) by different adsorbents

Adsorbents	Isotherm model	pH	Heavy metal Adsorption capacity (mg/g)		Ref.
			Cd(II)	Cr(VI)	
Magnetite NPs	Freundlich	6.98	3.00	4.65	Silva et al.(2014) [1]
Sunflower head carbon (SHC)	Langmuir	6	0.06	0.02	Jain et al.(2016) [2]
Sunflower stem carbon (SSC)	Langmuir	6	0.09	0.05	
Halomonas BVR1 immobilized in chitosan	Langmuir	6.8	23.88	—	Manasi et al.(2015) [3]
Chitosan	Langmuir	6.8	13.84	—	
HCB/TiO2	Langmuir	2	—	27.33	Zhang and Zhang(2014) [4]
Polyaniline grafted chitosan (PGC)	Langmuir	6	14.33	—	Karthik and Meenakshi(2015) [5]
Ferrihydrite (Fh)	Langmuir	4.5	—	12.97	Ni et al.(2016) [6]
Al-substituted Ferrihydrite (FAh)	Langmuir	4.5	—	39.79	
Chitosan-coated cotton gauze	Langmuir	3	—	12.40	Ferrero and Tonetti(2014) [7]
NaZ-nZVI	Langmuir	4.26	20.60	—	Tasharrofi et al.(2020) [8]
BC-SnZVI	Langmuir	5	32.55	43.10	Present study

Table S3 Assignment of the vibrational bands in the FTIR spectra

Functional groups	Peak position	Ref.
-OH	$3440cm^{-1}$	Wang et al. (2015) [9]
-COOH	$1383cm^{-1}$	Yang et al. (2018) [10]
C-O	$1119cm^{-1}$	Lyu et al. (2017) [11]
Fe-O	$600cm^{-1}$	Qu et al. (2021) [12]
Fe-S	$473cm^{-1}$	Yang et al. (2014) [13]

References

[1] M.J. Silva-Silva, O.F. Mijangos-Ricardez, V. Vázquez-Hipólito, S. Martinez-Vargas, J. López-Luna, Single and mixed adsorption of Cd(II) and Cr(VI) onto citrate-coated magnetite nanoparticles, Desalination Water Treat. 57(9) (2014) 4008-4017.

[2] M. Jain, V.K. Garg, K. Kadirvelu, M. Sillanpaa, Adsorption of heavy metals from multi-metal aqueous solution by sunflower plant biomass-based carbons, Int J Environ Sci Technol 13(2) (2016) 493-500.

[3] Manasi, V. Rajesh, N. Rajesh, An indigenous Halomonas BVR1 strain immobilized in crosslinked chitosan for adsorption of lead and cadmium, Int. J. Biol. Macromol. 79 (2015) 300-308.

[4] L. Zhang, Y.G. Zhang, Adsorption characteristics of hexavalent chromium on HCB/TiO2, Appl. Surf. Sci. 316 (2014) 649-656.
[5] R. Karthik, S. Meenakshi, Removal of Pb(II) and Cd(II) ions from aqueous solution using polyaniline grafted chitosan, Chem. Eng. J. 263 (2015) 168-177.
[6] C.Y. Ni, S. Liu, L.J. Cui, Z. Han, L. Wang, R.F. Chen, H. Liu, Adsorption performance of Cr(VI) onto Al-free and Al-substituted ferrihydrites, RSC Adv. 6(71) (2016) 66412-66419.
[7] F. Ferrero, C. Tonetti, M. Periolatto, Adsorption of chromate and cupric ions onto chitosan-coated cotton gauze, Carbohydr. Polym. 110 (2014) 367-373.
[8] S. Tasharrofi, Z. Rouzitalab, D.M. Maklavany, A. Esmaeili, M. Rabieezadeh, M. Askarieh, A. Rashidi, H. Taghdisian, Adsorption of cadmium using modified zeolite-supported nanoscale zero-valent iron composites as a reactive material for PRBs, Sci. Total Environ. 736 (2020) 139570.
[9] Z.Y. Wang, G.C. Liu, H. Zheng, F.M. Li, H.H. Ngo, W.S. Guo, C. Liu, L. Chen, B.S. Xing, Investigating the mechanisms of biochar's removal of lead from solution, Bioresour. Technol. 177 (2015) 308-317.
[10] F. Yang, S.S. Zhang, H.P. Li, S.S. Li, K. Cheng, J.S. Li, D.C.W. Tsang, Corn straw-derived biochar impregnated with alpha-FeOOH nanorods for highly effective copper removal, Chem. Eng. J. 348 (2018) 191-201.
[11] H. Lyu, J. Tang, Y. Huang, L. Gai, E.Y. Zeng, K. Liber, Y. Gong, Removal of hexavalent chromium from aqueous solutions by a novel biochar supported nanoscale iron sulfide composite, Chem. Eng. J. 322 (2017) 516-524.
[12] J. Qu, Y. Liu, L. Cheng, Z. Jiang, G. Zhang, F. Deng, L. Wang, W. Han, Y. Zhang, Green synthesis of hydrophilic activated carbon supported sulfide nZVI for enhanced Pb(II) scavenging from water: Characterization, kinetics, isotherms and mechanisms, J. Hazard. Mater. 403 (2021) 123607.
[13] R. Yang, Y. Wang, M. Li, Y.J. Hong, A New Carbon/Ferrous Sulfide/Iron Composite Prepared by an in Situ Carbonization Reduction Method from Hemp (Cannabis sativa L.) Stems and Its Cr(VI) Removal Ability, ACS Sustain. Chem. Eng. 2(5) (2014) 1270-1279.
